# Environmental impact on carcinogenesis under BRCA1 haploinsufficiency

**DOI:** 10.1186/s41021-023-00258-5

**Published:** 2023-01-13

**Authors:** Shinya Toyokuni, Yingyi Kong, Yashiro Motooka, Shinya Akatsuka

**Affiliations:** 1 Department of Pathology and Biological Responses, Nagoya University Graduate School of Medicine, 65 Tsurumai-Cho, Showa-Ku, Nagoya, 466-8550 Japan; 2 Center for Low-Temperature Plasma Sciences, Nagoya University, Furo-Cho, Chikusa-Ku, Nagoya, 464-8603 Japan

## Abstract

Cancer is the primary cause of human mortality in Japan since 1981. Although numerous novel therapies have been developed and applied in clinics, the number of deaths from cancer is still increasing worldwide. It is time to consider the strategy of cancer prevention more seriously. Here we propose a hypothesis that cancer can be side effects of long time-use of iron and oxygen and that carcinogenesis is an evolution-like cellular events to obtain “iron addiction with ferroptosis-resistance” where genes and environment interact each other. Among the recognized genetic risk factors for carcinogenesis, we here focus on *BRCA1* tumor suppressor gene and how environmental factors, including daily life exposure and diets, may impact toward carcinogenesis under BRCA1 haploinsufficiency. Although mice models of *BRCA1* mutants have not been successful for decades in generating phenotype mimicking the human counterparts, a rat model of *BRCA1* mutant was recently established that reasonably mimics the human phenotype. Two distinct categories of oxidative stress, one by radiation and one by iron-catalyzed Fenton reaction, promoted carcinogenesis in *Brca1* rat mutants. Furthermore, mitochondrial damage followed by alteration of iron metabolism finally resulted in ferroptosis-resistance of target cells in carcinogenesis. These suggest a possibility that cancer prevention by active pharmacological intervention may be possible for *BRCA1* mutants to increase the quality of their life rather than preventive mastectomy and/or oophorectomy.

## Introduction

Cancer is the leading cause of human mortality in Japan since 1981　(https://ganjoho.jp/public/qa_links/report/statistics/2022_en.html). Although numerous novel therapies, such as immune checkpoint inhibitors [1] and chimeric antigen receptor T-cell therapy [2], have been developed and applied in clinics recently, the number of deaths from cancer is still increasing worldwide (https://www.who.int/news-room/fact-sheets/detail/cancer). It is time to consider the strategies for cancer prevention more seriously and comprehensively to decrease the burden to the society.

We have been proposing a hypothesis that cancer can be the side effects of long-time use of iron and oxygen [3] if we can eliminate the established risks, including physical, chemical and biological carcinogens (https://www.who.int/news-room/fact-sheets/detail/cancer) and that carcinogenesis is generally a process to obtain “iron addiction with ferroptosis-resistance” [4]. The proof of concept for this hypothesis is that iron is indispensable for cell proliferation to replicate DNA [5] and that Fe(II) is a catalyst for the Fenton reaction, which generates the most damaging and mutagenic chemical species, hydroxyl radical [6]. This is further based on our own observation and observation by other investigators that 1) excess iron in various human pathology is associated with higher risk for carcinogenesis [7–9]; 2) iron reduction by phlebotomy decreases the cancer risk and mortality in a human intervention study [10]; 3) repeated iron-catalyzed Fenton reaction causes aggressive cancer that is similar to human counterparts not only in macroscopic/microscopic morphology but also in genetic alterations [11, 12]. These animal models include ferric nitrilotriacetate (Fe-NTA)-induced renal carcinogenesis [12–15] and asbestos-induced mesothelial carcinogenesis in rats [16–19]; 4) especially in the latter case, iron removal by iron chelating agent [20] or phlebotomy [21] can prevent mesothelial carcinogenesis to some extent. More detailed review on these topics are found elsewhere [9, 12, 22]. At first, we here describe the recent advances in iron metabolism in mammals, including the concept of ferroptosis.

### Recent advances in iron metabolism

Iron is the most abundant heavy metal in our body and is indispensable for all the lives on earth [7, 23, 24]. Iron basically works in two ways in higher mammals: 1) persistent electron transfer via redox cycling and 2) temporary oxygen storage as heme in hemoglobin, myoglobin, neuroglobin and cytoglobin. Indeed, ~ 60% of iron is in hemoglobin in humans. Because iron is thus important, our body is deficient of any active mechanism to discharge iron to outside our body [25].

**Fig. 1:**
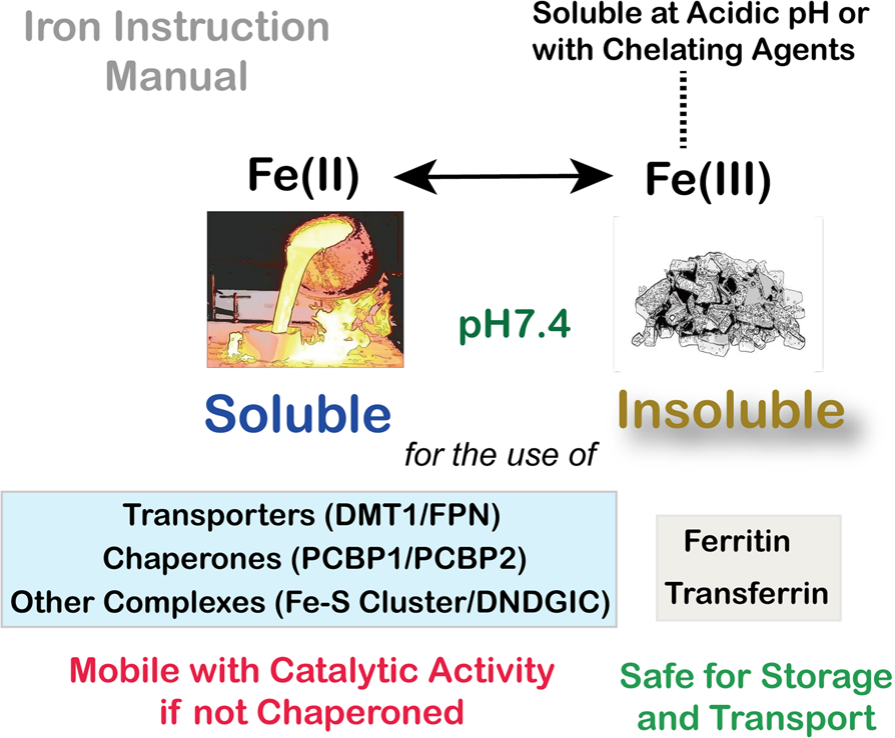
Difference in the biological significance of Fe(II) and Fe(III). DNDGIC, dinitrosyl-diglutathionyl-iron complex

**Fig. 2:**
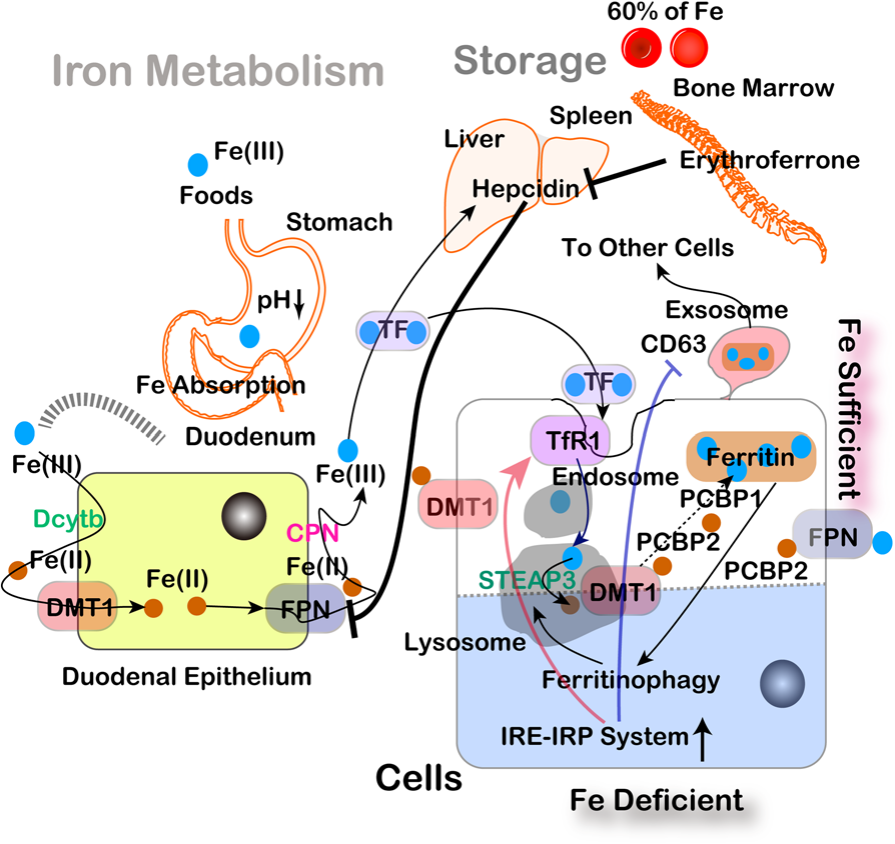
Current understanding of iron metabolism. Recently, many novel concepts have been established regarding iron metabolism, including ferritinophagy to take out iron from ferritin, cytosolic iron chaperones, PCBP1/2 and Fe(III)-loaded ferritin release via CD63-regulated exosomes. CPN, ceruloplasmin; Dcytb, duodenal cytochrome B; DMT1, divalent metal transporter-1 (SLC11A2); FPN, ferroportin (SLC40A1); TF, transferrin; STEAP3, six-transmembrane epithelial antigen of the prostate; TfR1, transferrin receptor-1; PCBP, poly(rC) binding protein; IRE-IRP, iron-responsive element-iron regulatory protein; brown circle as Fe(II); blue circle as Fe(III); green letter, reductase; pink letter, oxidase

Serum iron-transporting protein, transferrin, has been recognized since 1946 [26] and transferrin receptor 1 was identified in 1981 [26]. Iron storage protein ferritin was cloned in 1986 [27]. However, it took some extra time for membrane iron transporters and iron chaperones to be established [28, 29]. Of note, Fe(III) is insoluble at neutral pH and used for extracellular transport and intracellular storage (Fig. 1). In contrast, Fe(II) is soluble and used for transport across the membrane and intracellular transport. Labile iron is a concept indicating cytosolic mobile free iron [30], but some ambiguity still remains in that labile iron includes catalytic Fe(II), chaperoned Fe(II) by poly(rC) binding protein 1/2 (PCBP1/2) [31, 32] and dinitrosyl-diglutathionyl iron complex (DNDGIC) [33]. Figure 2 shows the current summary of iron metabolism.

A recent new finding in iron metabolism is that our cells use exosomes for the monopoly of iron inside ourselves [34]. The importance of iron for survival is the same for other microorganisms, such as bacteria, fungi and parasites. Those infectious agents try to steal iron from our cells. They use many different molecules, including siderophores [35]. Interestingly, one of the siderophores of a bacterium, desferrioxiamine, is used as an iron-chelating agent for medical use [36]. We recently found that a characteristic membrane surface molecule on exosome, CD63, is under the regulation of iron-responsive element/iron-regulatory protein (IRE/IRP) system [34]. This posttranscriptional regulatory system is specific for iron metabolism and IRE sequence is observed either in the 5’ or 3’ portion of mRNA of iron metabolism-associated genes. This is a system for iron deficiency (Fig. 2), considering the era of hunger [37]. Transferrin receptor 1 (Tfr1) mRNA has 5 IREs in the 3’ portions, thus increasing the lifetime of this message to increase the amounts of the Tfr1 protein. Conversely, translation of iron storage protein Fth1/Ftl is blocked for translation when the cell is iron-deficient. In the case of CD63, IRE sequence is present at the 5’ portion. If the cells harbor ample amounts of iron, this will be deblocked and exosomes with iron-loaded ferritin is generated through nuclear receptor activator 4 (NCOA4) and secreted toward the other cells of the same individual. Indeed, this is a safe strategy to transfer surplus iron to neighbor iron-deficient cells. Here we would like to stress that this IRE sequence in CD63 is present only in higher primates and is not present in mice or rats, which are used for experiments. However, this system is abused in asbestos-induced mesothelial carcinogenesis [22, 38, 39].

### Ferroptosis

**Fig. 3:**
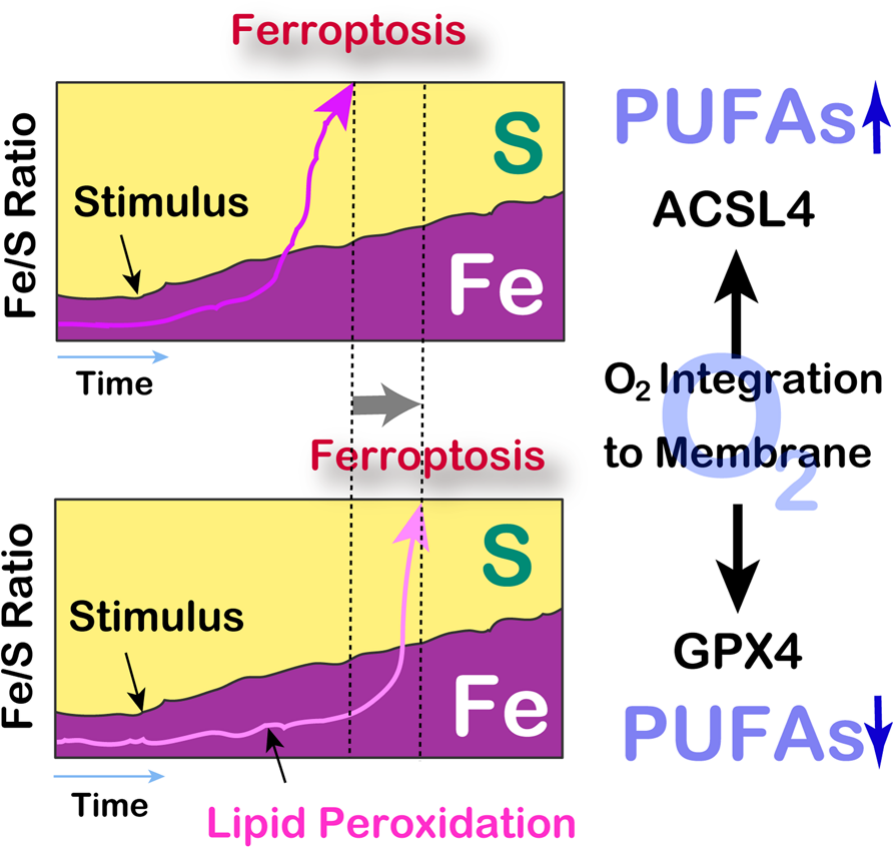
Current understanding of ferroptosis, catalytic Fe(II)-dependent regulated necrosis associated with lipid peroxidation. Ferroptosis is three dimensionally regulated by Fe, S and O. Transition to high Fe/S ratio by certain stimulus (*Ex.* excess iron and erastin [inhibitor of cystine/glutamate antiporter]) to cells initiate uncontrollable lipid peroxidation, which is cellular catalytic Fe(II) dependent and designated as ferroptosis. Ferroptotic cells reveal the morphology of necrosis. ACSL4, acyl-CoA synthatase long-chain 4; GPX4, glutathione peroxidase-4; PUFAs, polyunsaturated fatty acids

There are only two types of cell death classified by light microscopy, necrosis and apoptosis. However, starting from the 2000’s, many cell death modes were proposed, defined by the specific signaling pathways. These include ferroptosis (Fig. 3), catalytic Fe(II)-regulated necrosis accompanied by lipid peroxidation [40, 41]. Ferroptosis just celebrated its 10^th^ birthday in 2022, and this cell mode became popular evidenced by the exponentially increasing number of papers studying ferroptosis [42].

We have been working on iron-induced carcinogenesis for decades. Among them, repeated intraperitoneal injections of ferric nitrilotriacetate (Fe-NTA) induces renal cell carcinoma (RCC) in a high incidence (60 ~ 90%) in male rats [9, 12]. In this model, renal tubular necrosis is observed as early as 30 min in the proximal tubular cells with various lipid peroxidation products [43–46], which are the observation of our own in the 1980’s and 1990’s. When we first recognized the word ferroptosis in 2014, we immediately accepted it, based on our experience of this oxidative renal tubular damage. In the Fe-NTA-induced renal carcinogenesis model, renal tubular cells obtain ferroptosis-resistance in a few weeks after continued iron-catalyzed oxidative stress [12, 47, 48]. We have been proposing a hypothesis that carcinogenesis is a process to acquire ferroptosis-resistance under iron addiction via somatic mutation(s) [4]. Iron is an essential cofactor for ribonucleotide reductase for DNA synthesis and replication [49, 50], which is indispensable for proliferating cancer cells.

Accordingly, cancer cells harbor higher amounts of catalytic Fe(II) in the cytosol in comparison to the non-tumorous cells [51, 52]. This high amounts of catalytic Fe(II) is useful for DNA replication but also causes persistent oxidative stress to the cancer cells [53]. Thus, cancer cells are prepared to counteract this oxidative stress, for example, via the activation of Nrf2 transcription factor, a master regulator of antioxidative genes [54, 55]. Ferroptosis may be interpreted as relative predominance of iron over sulfur (sulfhydryls) by stimuli, which is modulated by the amounts of polyunsaturated fatty acids (PUFAs), mainly as phospholipids, in the cellular membrane (Fig. 3). This is indeed the Achilles’ heels of cancer cells and numerous ferroptosis inducers are currently under investigation for cancer therapy [3, 5, 56].

Alternatively, we recently found physiological ferroptosis. We selected a mouse monoclonal antibody for 4-hydroxy-2-nonenal (HNE)-modified proteins, HNEJ-1 clone, to detect ferroptotic cells in formalin-fixed paraffin-embedded specimens [57, 58]. Our present conclusion is that ferroptotic event occurs in nucleated red blood cells at E13.5 and aging cells of various organs in rats [59]. Thus, it is not strange to find ferroptosis in neuronal cells in various neurogenerative diseases [60–62]. Here researchers are trying to stop ferroptosis in the dying neuronal cells by developing agents to prevent ferroptosis. In summary, ferroptosis is now an optimal target for the development of new drugs both for induction and prevention.

### BRCA1

Current understanding is that cancer is a disease of the genome [63]. Thus far, we suggested that iron and oxygen can be the major mutagens in the long human lifetime of more than 80 years [3, 64]. Other than iron and oxygen, there are a plethora of mutagenic agents exposed to humans via skin, respiratory tract or gastrointestinal tract, which are both natural and industrial (https://monographs.iarc.who.int/agents-classified-by-the-iarc/). On the other hand, genetic susceptibility of each individual is as important as mutagens because there would be no carcinogenesis if the prevention and repair processes are perfect. Various familial cancer syndromes have been recognized from long time ago [63]. Since 1990’s, tumor suppressor genes were identified and cloned one by one [65]. These were the genes for the repair of various damage to genomic DNA or the genes to cause cell death with defined levels of biological/chemical/physical stimulus or damage. One of the most socially recognized tumor suppressor genes is BRCA1 due to the famous Hollywood actress, Angelina Jolie, known as the Angelina effect [66, 67].

Here according to a recent report on the Japanese population, the target organs for carcinogenesis of *BRCA1* mutants include female breast (odds ratio [OR], 16.1; p = 3.50 × 10^–11^) and ovary (OR, 75.6; p = 2.26 × 10^–22^) [68], which are critical for reproduction as the nutrient source of for the next generation and the reserve of oocytes, respectively. The present guideline still recommends prophylactic mastectomy [69] and oophorectomy [70] when necessary, which has been sensational to the general public. A higher risk for biliary tract cancer (OR, 17.4; p = 2.96 × 10^–7^), pancreatic cancer (OR, 12.6; p = 4.67 × 10^–5^) and gastric cancer (OR, 5.2; p = 3.40 × 10^–6^) is also noted recently for *BRCA1* mutants [68]. Considering the characteristics of target organs in BRCA1-associated carcinogenesis, we hypothesized that iron-associated oxidative stress may be in common as a promotional factor, especially for breast and ovary. This is based on the fact that both organs are deeply associated with iron metabolism including lactoferrin secretion in milk [71, 72] and ovulation. Ovarian endometriosis is closely associated with ovarian carcinoma through iron-mediated oxidative stress [73–76]. If so, some other preventive strategies may be possible.

### Species difference in animal experiment

*BRCA1* tumor suppressor gene was cloned in 1994 by Miki et al*.* [77]*.* Thereafter, hundreds of trials were performed to generate a feasible murine model of human *BRCA1* mutants. However, this was not successful in that heterozygous knockout of *BRCA1* alone showed no phenotype in carcinogenesis whereas homozygous knockout was embryonic lethal [78]. Many conditional knockout mice model was produced, but the results were negative. If the heterozygous knockout mice were crossed with *TP53*( ±) mice, the mice showed susceptibility to basal-like breast cancer [79].

However, it was surprising that rat *Brca1* mutant model (L63X/ +) shows the phenotype. This model was developed by Imaoka and Mashimo et al*.* in 2022 in Japan [80]. We believe that this is a species difference and that *Rattus norvegicus* is significantly closer to *Homo sapiens* in comparison to *Mus musculus*. We thus far observed similar phenomena in Fe-NTA-induced renal carcinogenesis. Whereas renal carcinogenesis is observed in mice and rats, phenotypes are quite different (Table 1), which is much milder in mice in comparison to rats [81].

**Table 1:** Species differences in ferric nitrilotriacetate (Fe-NTA)-induced renal cell carcinoma in rodents

Species	*Rattus norvegicus*	*Mus musculus*
First report	1982 [82]	1987 [14]
High susceptibility in males	Yes	Yes
Cancer incidence	high (72–92%) [13, 83]	low (7–62%) [84]
Strains used	*Wistar, BN, F-344, SD*	*A/J, C57/BL6*
Strain difference	None	Prominent [84]
Pulmonary metastasis	Common (~ 50%)	Very rare
Peritoneal invasion	Common	Rare
Histology	Renal cell carcinoma	Renal cell carcinoma
Tolerable Fe-NTA dose(ip)	~ 10 mg Fe/kg/day	3 ~ 7 mg Fe/kg/day
Genetic alterations by aCGH	Marked[11]	Less marked [84, 85]
*p16*^*Ink4a*^ deletion	Common [11, 86]	Rare [84, 85]

*BN* Brown-Norway, *F-344* Fischer-344, *SD* Sprague–Dawley

### BRCA1 and ferroptosis-resistance

We have recently applied Fe-NTA renal carcinogenesis model to male *Brca1*(L63X/ +) rats to evaluate whether iron-catalyzed oxidative stress [12] is important for *Brca1* mutant carcinogenesis [83]. The incidence of renal carcinogenesis was not changed between the *Brca1* mutant and the *wild-type*. However, the carcinogenesis was significantly promoted in the *Brca1* mutants by 3 months on average in comparison to the *wild-type*, which is a marked difference considering the average life time rats of ~ 3 y. This result indicates that iron-catalyzed oxidative stress is a promoting factor of carcinogenesis for *Brca1* mutants.

Furthermore, we found that renal cell carcinomas (RCCs) in *Brca1* mutants show more genomic alterations, including *c-Myc* amplification [83], which is indeed frequently observed in the breast carcinoma of human *BRCA1* mutants [87] and is a risk for poor prognosis [88, 89]. Here *c-Myc* amplification was often extrachromosomal. These results suggest that iron removal or avoidance of oxidative stress in the target organs could be an effective measure to prevent carcinogenesis in *BRCA1* mutants.

**Fig. 4:**
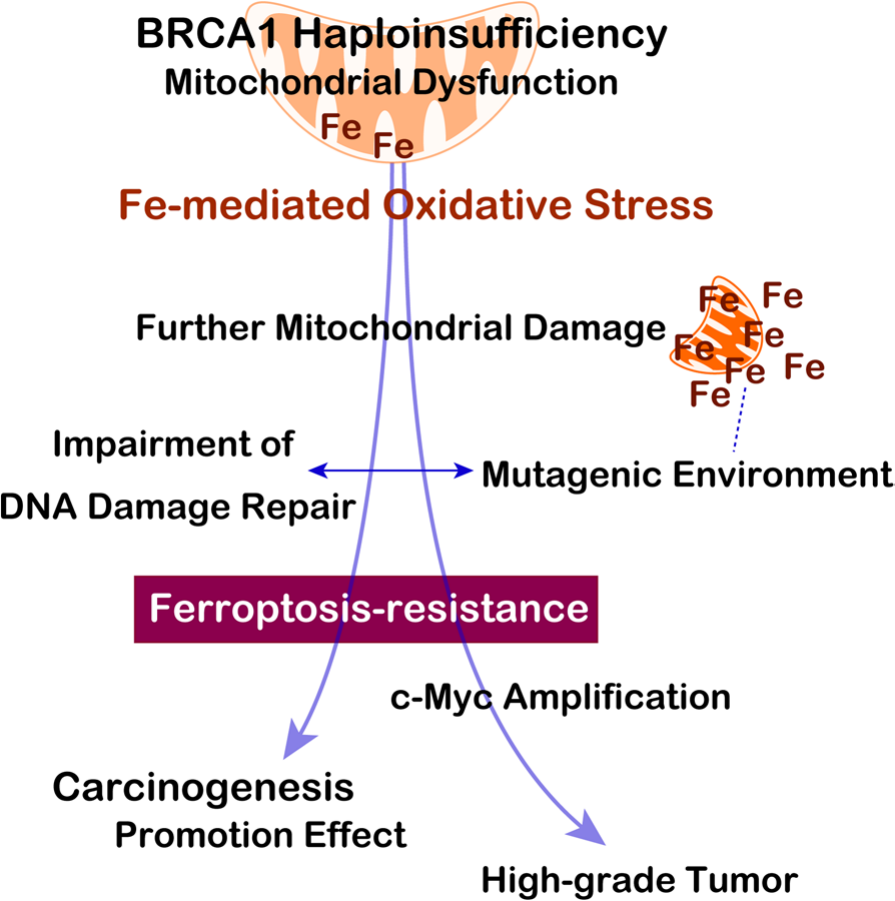
Role of BRCA1 haploinsufficiency in carcinogenesis. BRCA1 haploinsufficiency causes iron-rich mutagenic environment under persistent oxidative stress mediate by iron to promote carcinogenesis through ferroptosis-resistance and allows *c-Myc* amplification

We then undertook to understand the molecular mechanism why iron-catalyzed oxidative stress promotes renal carcinogenesis. We have performed expression microarray analysis in the subacute phase of 3 weeks during the renal carcinogenesis and found that higher mitochondrial damage is a key phenomenon [83]. Electron microscopical analysis revealed that even the untreated control kidney showed smaller and deformed mitochondria in the renal tubular cells of *Brca1* mutants. Since mitochondria play a central role in iron metabolism producing heme, it is plausible that mitochondrial damage alters iron metabolism in the entire cell, which produced a niche for carcinogenesis under mutagenic environment with Fe(III) abundance but with less catalytic Fe(II) at the subacute phase in the *Brca1* mutants in comparison to the *wild-type*. This is the mechanism how iron addiction with ferroptosis-resistance was generated (Fig. 4). We recently obtained similar results on chrysotile-induced malignant mesothelioma by the use of male *Brca1*(L63X/ +) rats [90]. However, we still need to know the role of Brca1 haploinsufficiency in this mitochondrial damage and the demonstration in human *BRCA1* mutant samples would be necessary.

## Conclusion

Cancer is basically a disease of the genome, where genome and environment persistently interact each other. We believe that even the long-use of iron and oxygen eventually causes various mutations, which may explain why aging stands as one of the highest risks for cancer. Some of the cancer susceptibility can be explained by the inactivation of tumor suppressor genes. Here this review article focused on how we undertook to find promoting factors in *BRCA1* mutants with a recently established rat *Brca1*(L63X/ +) model. During iron-induced renal carcinogenesis, *Brca1* haploinsufficiency allowed more genomic alterations, including amplification of *c-Myc*. Therefore, environmental factors, such as the control of iron and oxidative stress, may work as a strategy to prevent or delay carcinogenesis in *BRCA1* mutants.

## Data Availability

Not applicable.

## Abbreviations

DNDGIC: Dinitrosyl-diglutathionyl iron complex
Fe-NTA: Ferric nitrilotriacetate
HNE: 4-Hydroxy-2-nonenal
IRE/IRP: Iron-responsive element/iron-regulatory protein
NCOA4: Nuclear receptor activator 4
OR: Odds radio
PCBP1/2: Poly(rC) binding protein 1/2
PUFAs: Polyunsaturated fatty acids
RCC: Renal cell carcinoma
